# Automated quantification of left atrial size using three-beat averaging real-time three dimensional Echocardiography in patients with atrial fibrillation

**DOI:** 10.1186/s12947-015-0032-5

**Published:** 2015-08-25

**Authors:** Ran Heo, Geu-Ru Hong, Young-Jin Kim, Joel Mancina, In-Jeong Cho, Chi Young Shim, Hyuk-Jae Chang, Jong-Won Ha, Namsik Chung

**Affiliations:** Division of Cardiology, Severance Cardiovascular Hospital, Yonsei University College of Medicine, Seoul, Korea; Department of Radiology, Severance Hospital, Yonsei University College of Medicine, Seoul, Korea; Ultrasound Division, Siemens Medical Solution, Mountain View, CA, USA

**Keywords:** Three-dimensional echocardiography, Left atrial volume, Atrial fibrillation

## Abstract

**Background:**

Left atrial (LA) sizing in patients with atrial fibrillation (AF) is crucial for follow-up and outcome. Recently, the automated quantification of LA using the novel three-beat averaging real-time three dimensional echocardiography (3BA-RT3DE) is introduced. The aim of this study was to assess the feasibility and accuracy of 3BA-RT3DE in patients with atrial fibrillation (AF).

**Methods:**

Thirty-one patients with AF (62.8 ± 11.7 years, 67.7 % male) were prospectively recruited to have two dimensional echocardiography (2DE) and 3BA-RT3DE (SC 2000, ACUSON, USA). The maximal left atrial (LA) volume was measured by the conventional prolate-ellipse (PE) and area-length (AL) method using three-beat averaging 2D transthoracic echocardiography and automated software analysis (eSie volume analysis, Siemens Medical Solution, Mountain view, USA); measurements were compared with those obtained by computed tomography (CT).

**Results:**

Maximal LA volume by 3BA-RT3DE was feasible for all patients. LA volume was 68.4 ± 28.2 by PE-2DE, 89.2 ± 33.1 by AL-2DE, 100.6 ± 31.8 by 3BA-RT3DE, and 131.2 ± 42.2 mL by CT. LA volume from PE-2DE (R^2^ = 0.48, *p* < 0.001, ICC = 0.64, *p* < 0.001), AL-2DE (R^2^ = 0.47, *p* < 0.001, ICC = 0.67, *p* < 0.001), and 3BA-RT3DE (R^2^ = 0.50, *p* = 0.001, ICC = 0.65, *p* < 0.001) showed significant correlations with CT. However, 3BA-RT3DE demonstrated a small degree of underestimation (30.5 mL) of LA volume compared to 2DE-based measurements. Good-quality images from 3BA-RT3DE (*n* = 16) showed a significantly tighter correlation with images from CT scanning (R^2^ = 0.60, *p* = 0.0004, ICC = 0.76, *p* < 0.001) compared to those of fair quality.

**Conclusion:**

Automated quantification of LA volume using 3BA-RT3DE is feasible and accurate in patients with AF. An image of good quality is essential for maximizing the value of this method in clinical practice.

## Introduction

Left atrial (LA) size has been demonstrated as an important factor in atrial fibrillation (AF) development [1, 2]. In patients with a diagnosed AF, LA enlargement is related with a cerebrovascular outcome, [3] a risk of AF relapse after electrical cardioversion [4, 5] or catheter ablation [6, 7].

Therefore, accurate assessment of LA size is critical for making prognostic and treatment decisions in patients with AF. Transthoracic echocardiography (TTE) is the most common method for assessing LA volume [6]. However, data obtained from 2D echocardiography (2DE) are limited due to geometric assumptions and foreshortening of the LA cavity. LA remodeling is frequently asymmetrical, rendering standard geometric assumptions even more inadequate [8]. Therefore, three-dimensional assessment of LA might help to solve this issue. In addition, current recommendations suggest multi-beat measurements of LA volume in AF patients, which further increases the potential variability of 2DE images [9, 10].

Three-dimensional echocardiography (3DE) exhibits accurate assessment of LA volume and better intra-observer and inter-observer agreement when compared to those obtained with magnetic resonance imaging (MRI) [11, 12] or computed tomography (CT) [13, 14]. However, previous studies often excluded AF patients [11, 14–17]. Recently, a novel automated three-beat averaging real-time three-dimensional echocardiography (3BA-RT3DE) method was introduced [18]. In this study, we examined the feasibility and accuracy of the 3BA-RT3DE to measure LA volume in patients with stable AF compared to that of 2DE and CT.

## Materials and methods

### Study population

Thirty-one consecutive patients with AF were prospectively recruited. All patients were stable without any changes in clinical condition or treatment between studies. Inclusion criteria were ECG-documented AF with an echo window that was at least fair in 2DE. Exclusion criteria included decompensated heart failure, tachycardia (heart rate over 100 beats per minute), prior valve replacement surgery, and history of renal failure. Patients received 2DE, 3BA-RT3DE, and cardiac 64-slice multi-detector CT scans. The median interval between CT and echocardiography was two days (IQR 0–7 days). And 74.2 % of CT scans were done within one week. Informed consent was obtained from all patients, and the institutional review board of Severance Hospital of Yonsei University approved the study protocol.

### Echocardiographic image acquisition and quantification

**Fig. 1:**
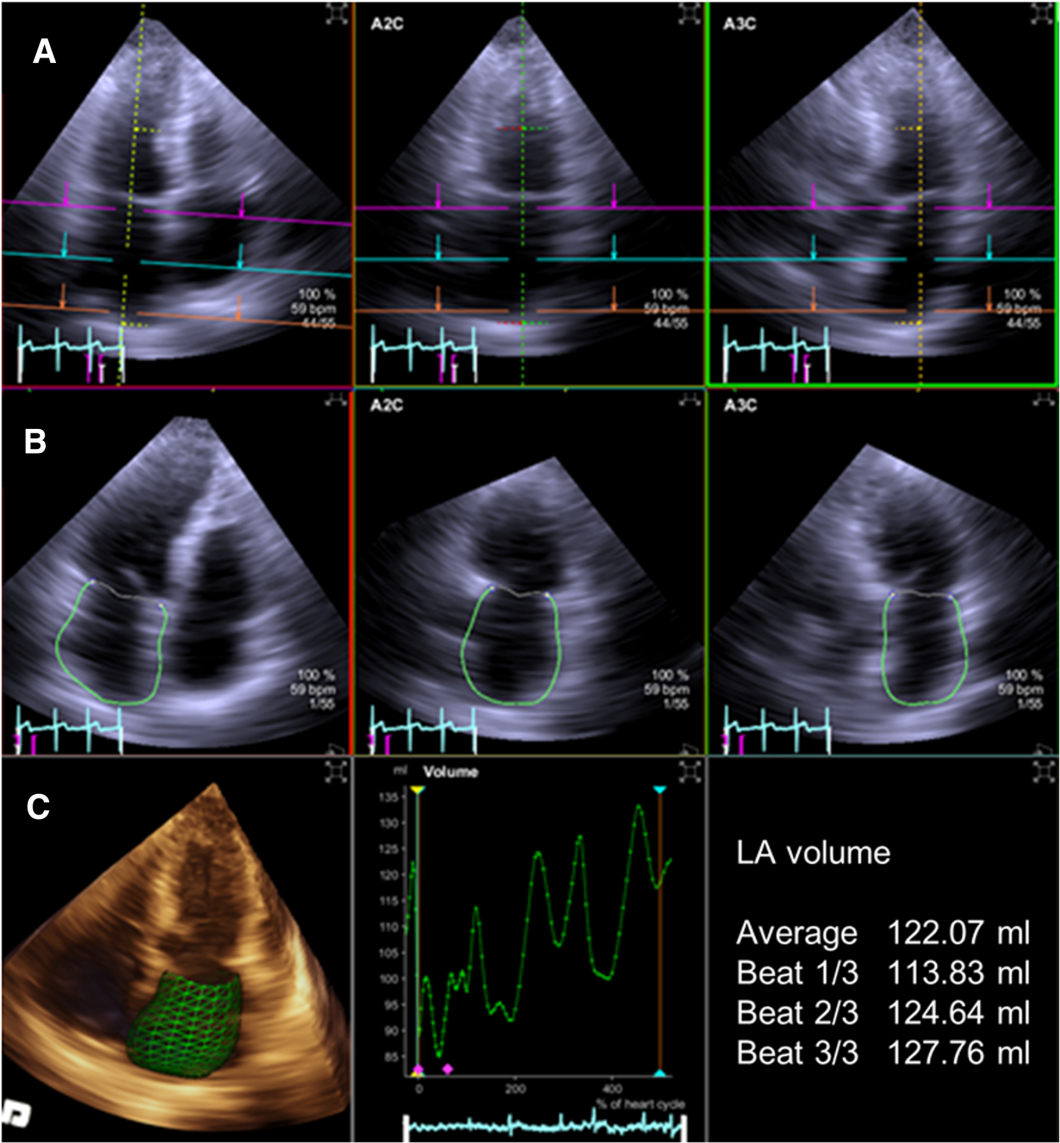
Measurement of left atrial volume by three-beat averaging real-time three-dimensional echocardiography with semi-automated endocardial border detection. **a** Set the region of interest in the left atrium. **b** Perform semi-automated endocardial border tracking of the left atrium. **c** Set left atrial volume curve, and calculate average left atrial LA volume from three consecutive beats

Two-dimensional echocardiography was performed with commercially available ultrasound systems (Philips IE 33 (Philips Andover, Andover, MA, USA), GE vivid E9 (GE Healthcare, Fairfield, CT, USA), ACUSON Sequoia C512 (Siemens Germany, North Rhine-Westphalia, Germany)). LA volumes were assessed by 2DE and RT3DE with all patients in the left lateral decubitus position using gray-scale, second-harmonic, two-dimensional imaging; image contrast, frequency, depth, and sector size were adjusted for an adequate frame rate and optimal LA border visualization. Parameters related to LA volume were obtained in parasternal long, apical 2- and 4-chamber views by 2DE. All 2DE measurements were performed via averaging three beats at relatively regular rhythms, aiming to obtain maximal LA volume at end-systole [9]. The prolate-ellipse (PE) and area-length (AL) methods were used to quantify 2DE LA volume measurements [9]. The PE method used the formula V = 0.523 × (D1) × (D2) × (D3), where D1 was measured from the middle of the plane of the mitral annulus to the superior aspect of the left atrium in a four-chamber view, D2 was the orthogonal short-dimension to D1, and D3 reflected the anterior-posterior diameter measured in a parasternal long-axis. The AL method used the formula 8/π × A1 × A2/L, where A1 was the left atrial area from the apical four-chamber view, and A2 was measured from the apical two-chamber view. L was the shortest superior-inferior diameter measured in the apical four- or two-chamber view. For 3BA-RT3DE measurements, LA volume was acquired during three consecutive beats with the Siemens ACUSON SC2000, 4Z1c real-time volume imaging transducer (2.5 MHz). Images by 2DE and 3DE were acquired during a single examination. Real-time 3D apical full-volume images were acquired. All image data were analyzed using eSie analysis software which is an offline, dedicated SC2000 workplace system (Siemens Medical Solution, Mountain View, CA, USA). Automated border tracking of LA and computed maximal LA volume without geometric assumptions were performed as shown in Fig. 1. The software created a geometric model of LA through manual designation of the mitral annulus and superior dome point in the frames corresponding to end-diastolic and end-systolic time; subsequent border detection was performed based on an automated algorithm that detected the endocardial wall interface. This automated identification was based on pattern recognition learning from large annotated data repositories. This technology also allows for automated delineation (auto-contouring) of the endocardium of the LA throughout the entire cardiac cycle. In the following step, the contours were manually corrected if necessary. The pulmonary vein orifices and LA appendage were not included in the contour. Image quality from RT3DE was stratified as good or fair. Good quality was defined as those images with more than 80 % of LA borders visible, and fair quality images had more than 60 % of LA borders visible [19].

Two experienced cardiologists participated in the analysis of 3BA-RT3DE. Each observer had analyzed 30 cases each before analyzing the patients in this study. Intra- and inter-observer agreement for 3BA-RT3DE was assessed using repeated measurements of all patients. The second observer used the same data sets for offline analysis as the first observer but was blinded to the results or identities of the subjects. Both observers were blinded to the results from other modalities.

### CT imaging protocol

Cardiac CT scans were performed using a dual-source CT scanner (SOMATOM Definition Flash; Siemens Healthcare, Forchheim, Germany) during a single breath-hold. Imaging protocol included the administration of beta-blocker, if the baseline heart rate was above 65 beats per minute, if contraindications were absent. Heart rates during acquisition did not differ significantly between echocardiography (mean, 76.9 ± 20.1 beats/min; range, 42–120 beats/min) and CT (mean, 65.7 ± 16.6 beats/min; range, 41–136 beats/min). Optimal delay times for maximal LA volume were determined after a bolus injection of 10 ml of iopamidol (Pamiray; 370 mg of iodine/ml, Dongkook Pharma, Seoul, Korea) followed by 20 ml of saline at 5 ml/s. All CT scans were performed using the triple-phase injection method (70 ml of iomadidol followed by 30 ml of 30 % blended iopamidol with saline and 20 ml of saline at 5 ml/s). For atrial volume measurement, ECG-gated axial acquisition targeting end-systolic phase using the absolute delay method (a fixed time delay after the R wave) or maximal LA volume was utilized. Tube voltage and tube current were chosen using automatic tube potential selection software (Care kV; Siemens Healthcare, Forchheim, Germany). Images were reconstructed with a slice thickness of 0.75 mm and a reconstruction increment of 0.5 mm using sonogram-affirmed iterative reconstruction (SAFIRE; Siemens Healthcare, Forchheim, Germany) technique. CT images were uploaded into volume-rendering software (Aquaris iNtuition Edition V4.4.11, TeraRecon, San Mateo, CA, USA) and LA volume was automatically or semi-automatically segmented on the basis of the 3-dimensional threshold method, and atrial volumes were obtained after the manual exclusion of the pulmonary veins and vena cava as described in previous studies [20, 21].

### Statistical analyses

Normality of continuous variables was assessed by the Kolmogorov-Smirnov test. Continuous data are presented as mean ± standard deviation. Categorical data are expressed as absolute numbers or percentages. Independent *t*-test was used to compare subgroups. Paired *t*-test was performed to compare values from two methods. Linear regression analysis was performed to evaluate relationships between methods. Values from different techniques were compared using intra-class correlation coefficient (ICC). Bland-Altman analysis was performed to evaluate differences in maximal LA volume between different techniques. Inter-observer variability was assessed by Bland-Altman analysis and ICC. A P value < 0.05 was considered statistically significant. SPSS version 16 (SPSS, Inc., Chicago, IL) was used for statistical testing.

## Results

The mean age of patients was 62.8 ± 11.7 years, and there were more males (Table 1). Quantification of maximal LA volume by 3BA-RT3DE was feasible in all 31 patients. Images of good quality were obtained in 16 patients; the remaining 15 patients had images of fair quality. The mean volume rate was 18.1 ± 2.8 volume per second.

**Table 1:** Baseline characteristics of patients

Variables	Overall	Fair quality	Good quality	*P* value
	(*n* = 31)	(*n* = 16)	(*n* = 15)	
Age(y)	62.8 ± 11.7	66.5 ± 10.5	58.8 ± 11.8	0.90
Male gender (%)	21 (67.7)	10 (62.5)	11 (73.3)	0.901
Height (cm)	166.6 ± 8.7	166.0 ± 7.9	167.2 ± 9.7	0.254
Weight (kg)	65.3 ± 13.3	63.1 ± 13.5	67.7 ± 13.0	0.912
Systolic BP (mmHg)	129.0 ± 20.1	133.4 ± 21.8	124.4 ± 17.7	0.062
Diastolic BP (mmHg)	78.7 ± 12.9	77.4 ± 13.3	80.1 ± 12.8	0.994
Heart rate (bpm)	76.9 ± 20.1	75.9 ± 20.2	77.9 ± 20.6	0.851
LVEDD (mm)	49.9 ± 5.4	48.6 ± 4.2	51.2 ± 6.3	0.140
LVESD (mm)	34.0 ± 6.2	32.9 ± 4.0	35.1 ± 7.9	0.179
LV EF (%)	61.9 ± 11.0	61.9 ± 8.3	61.8 ± 13.6	0.343

Data are expressed as number (%) or mean ± standard deviation

*BP* blood pressure, *LVEDD* left ventricular end-diastolic diameter, *LVESD* left ventricular end-systolic diameter, *LV EF* left ventricular ejection fraction

**Fig. 2:**
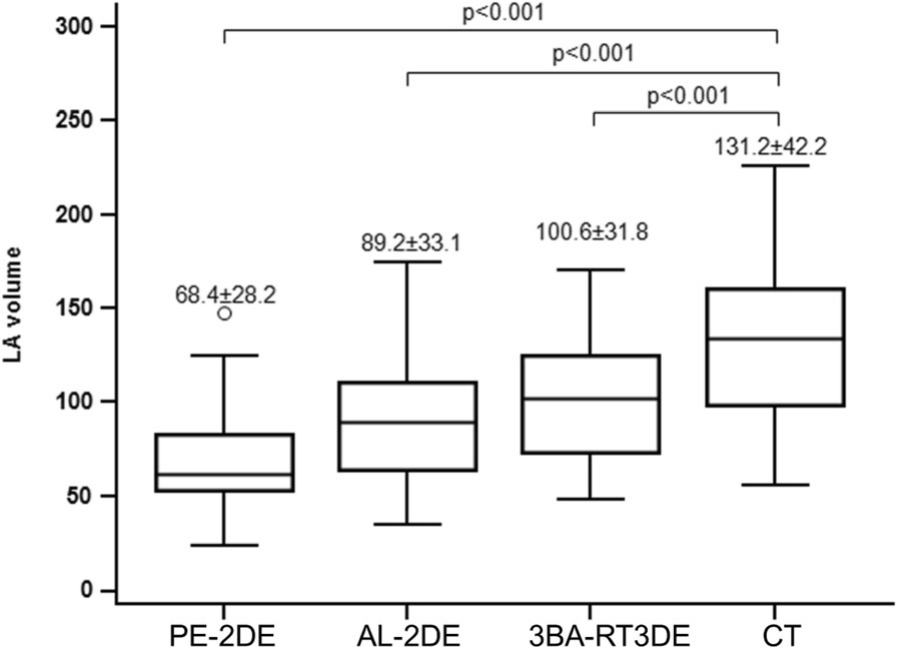
Left atrial volume measurements from each modality. Values are mean ± standard deviation. AL = area-length method, CT = computed tomography, LA = left atrium, PE = prolate-ellipse method, 3BA-RT3DE = three-beat averaging real-time three-dimensional echocardiography, 2DE = two dimensional echocardiography

LA volume was underestimated by echocardiography-based modalities compared to CT. However, 3BA-RT3DE showed the lowest degree of underestimation of maximal LA volume compared to that from CT. The maximal LA volume was 68.4 ± 28.2, 89.2 ± 33.1, 100.6 ± 31.8, and 131.2 ± 42.2 mL from PE-2DE, AL-2DE, 3BA-RT3DE, and CT, respectively (Fig. 2). LA volume by 3BA-RT3DE was significantly greater than (those obtained by) both PE-2DE (*P* < 0.001) and AL-2DE (*p* = 0.02).

**Fig. 3:**
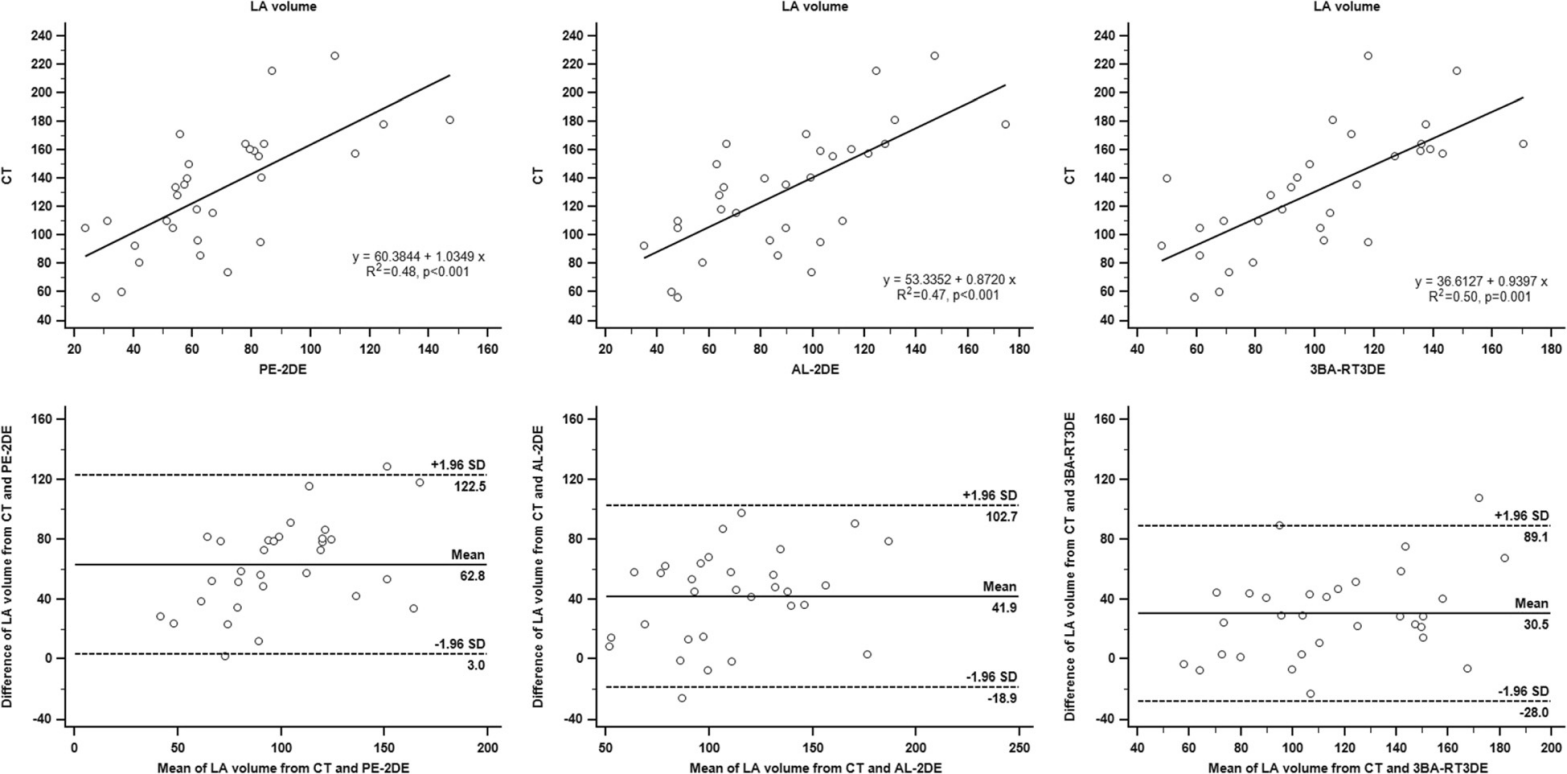
Relationship between measurements obtained by two-dimensional echocardiography (prolate-ellipse and area-length methods), three-beat averaging real-time three-dimensional echocardiography, and computed tomography. AL = area-length method, CT = computed tomography, LA = left atrium, PE = prolate-ellipse method, 3BA-RT3DE = three-beat averaging real-time three-dimensional echocardiography, 2DE = two dimensional echocardiography

The correlation between the maximal LA volume obtained from CT was fair with 2DE-based methods regardless of PE-2DE (R^2^ = 0.48, *p* < 0.001, ICC = 0.64, *p* < 0.001) or AL-2DE (R^2^ = 0.47, *p* < 0.001, ICC = 0.67, *p* < 0.001). The data from 3BA-RT3DE showed similar correlation (R^2^ = 0.50, *p* = 0.001, ICC = 0.68, *p* < 0.001) with measurements obtained by CT scan (Table 2, Fig. 3). The mean difference in the maximal LA volume measured by PE-2DE, AL-2DE, and 3BA-RT3DE and that from CT was 62.8, 41.9, and 30.5 mL, respectively. The limits of agreement for the 3BA-RT3DE were similar among echocardiography-based methods (1.96SD: 59.7 mL for PE-2DE, 60.8 for AL-2DE, 58.6 mL for 3BA-RT3DE). The difference between maximal LA volume by echocardiographic method and CT increased as the LA became enlarged. This trend was similar in 2DE and 3BA-RT3DE images (Fig. 3).

**Table 2:** Maximal left atrial volume (mL) using different modalities and comparison of it by echocardiography to that by computed tomography

Modality	Mean ± SD	Correlation with maximal LA volume by CT			
		R^2^	*P* value	ICC	*P* value
PE-2DE	68.4 ± 28.2	0.48	<0.001	0.64	<0.001
AL-2DE	89.2 ± 33.1	0.47	<0.001	0.67	<0.001
3BA-RT3DE	100.6 ± 31.8	0.50	0.001	0.65	<0.001
CT	131.2 ± 42.2				

*AL* area-length method, *CT* computed tomography, *ICC* intra-class correlation coefficient, *LA* left atrium, *PE* prolate-ellipse method, *3BA-RT3DE* three-beat averaging real-time three-dimensional echocardiography, *SD* standard deviation, *2DE* two dimensional echocardiography

**Fig. 4:**
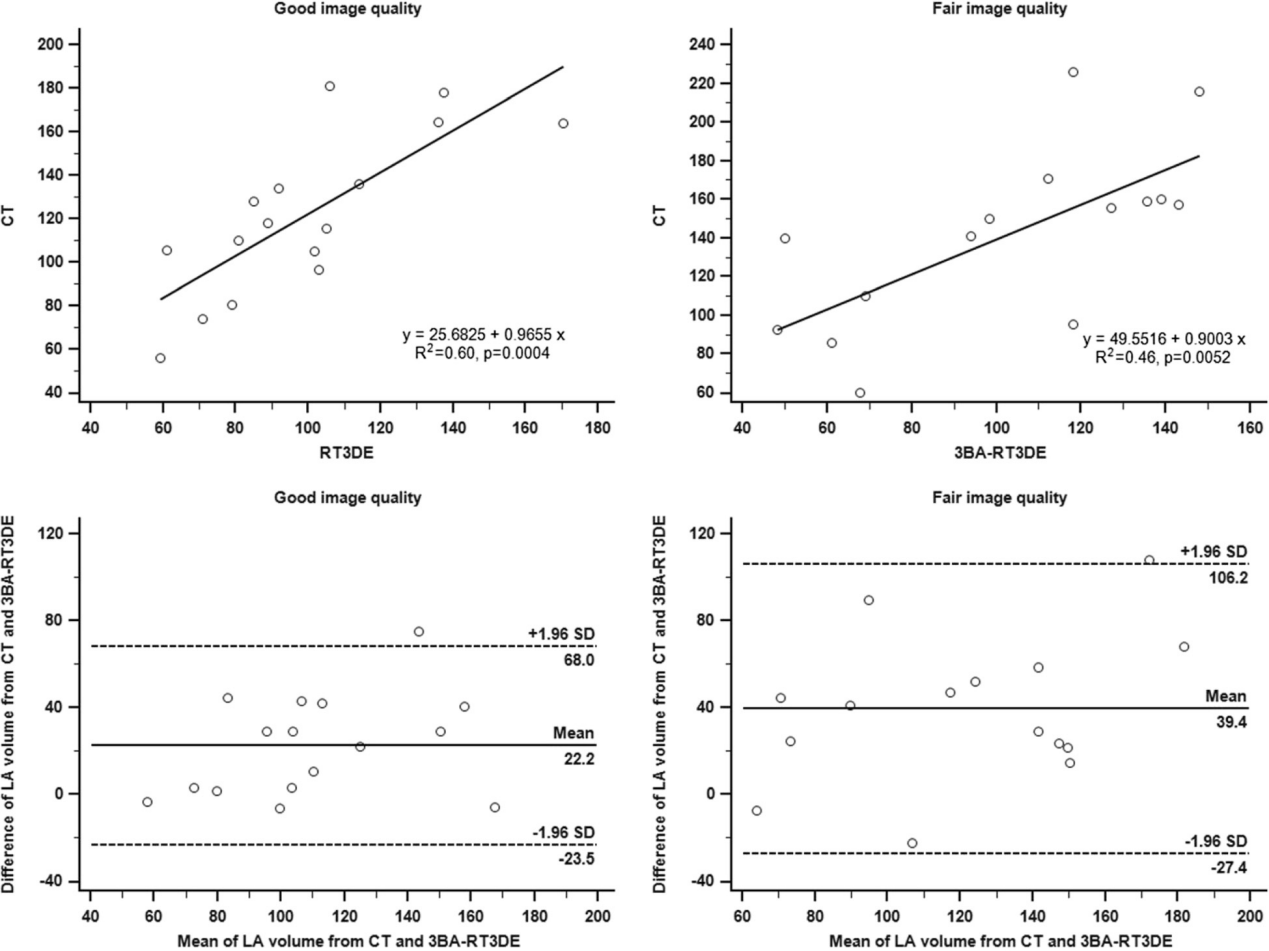
Relationship between measurements obtained by three-beat averaging real-time three-dimensional echocardiography and computed tomography in good (*left panel*) and fair (*right panel*) image quality groups. CT = computed tomography, LA = left atrium, 3BA-RT3DE = three-beat averaging real-time three dimensional Echocardiography

Images from 3BA-RT3DE were divided into two groups according to image quality. The maximal LA volume of the good-quality group (*n* = 16) showed a significantly better correlation between 3BA-RT3DE and CT (R^2^ = 0.60, *p* = 0.0004, ICC = 0.76, *p* < 0.001). In contrast, the fair-quality group showed a relatively poor correlation (R^2^ = 0.46, *p* = 0.0052, ICC = 0.65, *p* = 0.003) (Table 3). Furthermore, the good-quality group showed a consistent difference in LA volume between 3BA-RT3DE and CT across the entire range of LA volumes (Fig. 4). In addition, the limits of agreement were narrower for the good-quality group compared to that of the fair-quality group (1.96SD: 45.8 mL for good quality group, 66.8 mL for fair quality group).

**Table 3:** Comparison of maximal left atrial volume (mL) using real-time three-dimensional echocardiography in good and fair image quality group

Image quality	Mean ± SD	Correlation with maximal LA volume by CT			
		R^2^	*P* value	ICC	*P* value
Good	99.4 ± 29.6	0.60	0.0004	0.76	<0.001
Fair	101.9 ± 35.0	0.46	0.0052	0.66	0.003

*CT* computed tomography, *ICC* intraclass correlation coefficient, *LA* left atrium, *SD* standard deviation

**Fig. 5:**
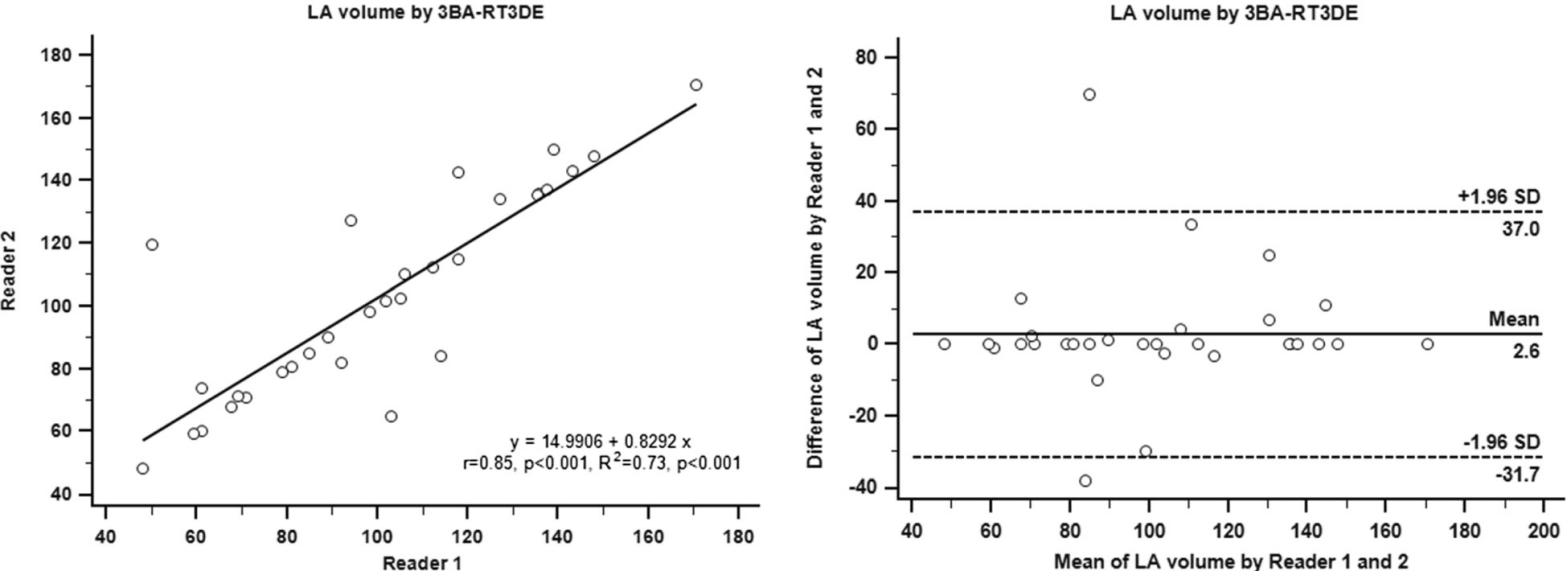
Inter-observer agreement of left atrial volume by three-beat averaging real-time three-dimensional echocardiography. LA = left atrium, 3BA-RT3DE = three-beat averaging real-time three-dimensional echocardiography

Intra- and inter-observer agreement of 3-beat averaging RT3DE in maximal LA volume was assessed. It showed good intra- (r = 0.95, *p* < 0.001, R^2^ = 0.90, *p* < 0.001, ICC = 0.95, *p* < 0.001) and inter-observer agreement (r = 0.85, *p* < 0.001, R^2^ = 0.73, *p* < 0.001, ICC = 0.85, *p* < 0.001). The mean inter-observer difference of 3-beat averaging RT3DE was 2.6 mL (Fig. 5).

## Discussion

In this study, a novel automated 3BA-RT3DE was feasible in all patients and showed fair correlation and a lower degree of underestimation compared to 2DE, with CT scanning as the comparison modality. Furthermore, in images of good quality, maximal LA volume measured by 3BA-RT3DE showed better correlation and less variability compared to that of images with fair quality.

Many studies have compared LA volume by 3DE with 2DE, [22] CT [13, 14] or MRI [11, 12, 17, 23]. These published results show that 3DE yields reduced variability and higher or similar accuracy to that of 2DE, mainly because 3DE addresses some of the limitations of 2DE, such as geometric assumption [22, 24–27]. Moreover, 3DE measurements of LA volumes have clinical value for assessing response to therapy and for predicting clinical events in patients with severe left ventricular dysfunction [24, 28, 29]. However, consistent underestimation was shown in most studies, because of ambiguous endocardial borders produced by apical imaging in the far field of the ultrasound beam. Previous studies using 3DE for LA volume quantification used a semi-automated endocardial tracking algorithm that was originally developed for the quantification of left ventricular size and function [14, 22, 23]. However, this 3DE direct volumetric algorithm was validated by MRI and has good comparability to the reference modality [30]. One study used RT3DE with software dedicated to the analysis of LA volume and demonstrated similar results to those of our study [31]. In that study, LA volume was more accurately determined with RT3DE than 2DE, and there was a trend toward increased bias in patients with enlarged atria. However, AF patients were excluded from that study, and manual endocardial tracing was required [31].

Recently, two studies were conducted in AF patients to validate the RT3DE-based LA volume quantification with CT [32] or MRI [12] as a reference method. Rohner et al. showed LA volumes and ejection fraction as assessed by RT3DE compared to those obtained by CT. Although RT3DE showed a trend toward underestimating LA volume, it correlated strongly with CT measurements. Furthermore, there was robust inter- and intra-observer variability [32]. However, 85.3 % of patients were described as being in sinus rhythm on baseline, and it only used one-beat RT3DE for LA measurement, which is not concordant with the guideline. In the other study, 2DE and RT3DE were compared with MRI for abilities to assess LA volume. RT3DE showed a moderate improvement in accuracy and the narrowest limits of agreement compared to that of 2DE; further, LA volume was underestimated by echocardiography-based methods compared with MRI. However, it also selected single-beat for evaluation of LA volume [12]. In our study, echocardiography-based measurements of maximal LA volume showed significant correlations and similar degrees of underestimation compared with those from CT, as previously published [32]. However, 3BA-RT3DE demonstrated the lowest degree of underestimation and the narrowest limit of agreement. Furthermore, we demonstrated that image quality is an important factor for LA volume assessment by 3DE in patients with AF. The correlation and degree of underestimation was significantly higher in images of good quality compared with images of fair quality. This is an important consideration for clinical practice, as many patients with AF exhibit poor or fair echo windows. Thus, RT3DE may be the appropriate method for measuring LA volume in patients with AF. The other potential explanation for LA volume underestimation is that LA volume may be overestimated by CT. A recent study by Agner et al. investigated LA volume by volumetric 2DE, CT, and MRI. In that study, CT overestimated LA volume compared to MRI in patients with permanent AF, whereas TTE significantly underestimated LA volume compared to CT and MRI [33]. This difference might be related to volume effects of the contrast and saline chaser. In addition, the administration of a beta blocker to control heart rate might affect LA volume. Furthermore, although CT and MRI showed excellent intra- and inter-observer agreements, these might not be practical approaches for daily clinical practice. Currently, MRI takes approximately 60 min for image acquisition. CT scanning exposes patients to radiation regardless of the dose. The risk of renal injury is also high in patients with AF, as they often have renal disease. Patients with AF should be followed regularly for LA volume assessment; for this purpose, echocardiographic methods are widely available. However, the current recommendation of measuring at least 5 beats for LA volume is rather time-consuming. Thus, 3BA-RT3DE is a promising tool for LA assessment in AF patients, particularly for images of good quality. Further studies in larger populations of patients with diverse pathologies will allow the range of applicability of 3DE-based LA volume quantification to be carefully assessed.

### Limitations

Our study has limitations. We only examined maximal LA volume, which is strongly supported as a metric for assessing cardiovascular risk. However, some studies suggest that other parameters, such as minimal LA volume and phasic changes of LA, are also related to cardiovascular prognosis [28, 34–37]. These parameters might provide incremental information. In addition, although 3BA-RT3DE showed improved accuracy for LA volume measurements compared to 2DE, the clinical significance remains unclear due to the lack of prognostic information. Thus, further studies are needed to understand the prognostic value of RT3DE-based LA assessment in AF patients. Moreover, we used beta-blocker during the CT scan, which might have affected the hemodynamics of LA volume. However, our population was composed of AF patients; hence, the heart rate variability and potential use of beta-blocker were innate issues of these patients.

## Conclusion

Automated quantification of LA volume using 3BA-RT3DE is feasible and accurate in patients with AF. An image of good quality is essential for maximizing the value of this method in clinical practice. This technique might advance and enhance the integration of RT3DE into routine clinical practice.
